# *Toxoplasma gondii*-induced host’s high secretion of 25-hydroxycholesterol for immunoprotection

**DOI:** 10.1186/s13071-025-06890-0

**Published:** 2025-08-04

**Authors:** Zi-Han Yang, Wei-Ling Wu, Jia-Jia Zheng, Chi Zhang, Ying-Ying Lu, Hong-Juan Peng

**Affiliations:** Department of Pathogen Biology, Guangdong Provincial Key Laboratory of Tropical Diseases Research, School of Public Health; Key Laboratory of Infectious Diseases Research in South China (Southern Medical University), Ministry of Education, Southern Medical University, 1023-1063 South Shatai Rd, Guangzhou, 510515 Guangdong People’s Republic of China

**Keywords:** Toxoplasma gondii, Metabolomics, Cholesterol metabolism, 25-hydroxycholesterol, Microglia, Transcriptomics

## Abstract

**Background:**

*Toxoplasma gondii*, a parasitic protozoan affecting approximately one-third of global population, causes opportunistic toxoplasmosis. It penetrates barriers to immune-privileged sites, causing encephalitis, retinochoroiditis, and fetal damage. The infection may be linked to neurodegenerative and psychiatric disorders. The *T. gondii*–host interaction mechanism remains central to understanding its pathogenesis. The changes in small molecule metabolites after infection, which affects the central nervous system (CNS) normal function, have been poorly characterized.

**Methods:**

The metabolic alterations in brain tissues of sv129 mice infected by *T. gondii* at 9 days post-infection (DPI) were analyzed through untargeted metabolomic detection. Cholesterol metabolic reprogramming was assessed through analysis of related gene’s transcription with quantitative reverse transcription polymerase chain reaction (qRT-PCR). The primary target cells responsible for cholesterol metabolic dysregulation were identified through detection of the secreted cytokines with enzyme-linked immunosorbent assay (ELISA). The *T. gondii* replication in host cells treated with 25-HC was evaluated using immunofluorescence assay (IFA). Transcriptomic analysis was performed to identify the differentially expressed genes (DEGs) in the host cells infected by *T. gondii* and/or treated with 25-HC, and the host cell M1 polarization was confirmed by qRT-PCR.

**Results:**

Brain metabolomic profiling identified 19 differentially expressed metabolites (including 25-HC), primarily involved in amino acid metabolism and cholesterol metabolism pathways (biosynthesis of primary bile acids and steroids). *Toxoplasma gondii* infection triggered host cholesterol metabolic reprogramming and promoted 25-HC secretion from glial cells, which indirectly inhibited *T. gondii*’s proliferation in host cells. Transcriptomic analysis revealed that 25-HC upregulated the expression of chemokines, C-type lectin receptors, and inflammation-related genes. Notably, 25-HC was verified to confer host resistance against *T. gondii* infection by promoting microglial M1 polarization.

**Conclusions:**

Our study demonstrated that *T. gondii* infection activates the CH25H-25-HC axis to induce microglial M1 polarization and cytokine secretion, thereby establishing an anti-*Toxoplasma* defense. These findings highlight the central role of cholesterol metabolism in *T. gondii* pathogenesis and provide innovative strategies for the diagnosis, prevention, and treatment of toxoplasmosis.

**Graphical Abstract:**

**Figure:**
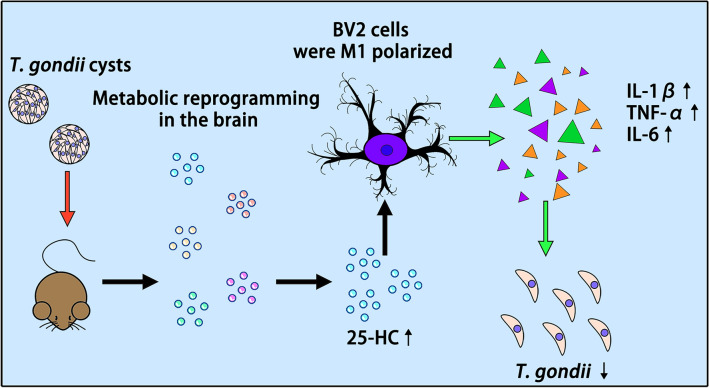

**Supplementary Information:**

The online version contains supplementary material available at 10.1186/s13071-025-06890-0.

## Background

*Toxoplasma gondii* is an obligate intracellular parasitic protozoan that causes opportunistic infections, affecting approximately 30–50% of the global population [1]. The primary route of *T. gondii* infection is oral ingestion, where humans become infected through consumption of raw or undercooked meat containing tissue cysts or pseudocysts, or through congenital transmission [2]. *Toxoplasma gondii* can cross various human biological barriers, including the blood–brain barrier (BBB), blood–ocular barrier, and placental barrier, reaching immune-privileged sites such as the brain, retina, and placenta [3]. Though most of *T. gondii* infections in immunocompetent individuals are asymptomatic, the infection in immunocompromised patients can lead to severe conditions including encephalitis and retinochoroiditis, and primary infection during pregnancy can result in adverse pregnancy outcomes [4, 5].

*Toxoplasma gondii* is a neurotropic parasite associated with various neurodegenerative and neuropsychiatric disorders, including schizophrenia, anxiety disorders, bipolar disorder, and depression [6–8]. Following infection of the central nervous system (CNS), microglia and astrocytes become hyperactivated, releasing excessive proinflammatory cytokines such as tumor necrosis factor-alpha (TNF-α) and interferon-gamma (IFN-γ) [9]. This triggers neuroinflammation and neuronal apoptosis, with the severity of pathological changes increasing in a time-dependent manner [9]. Microglia can resist pathogen infection through releasing proinflammatory cytokines such as IFN-γ and interleukin-1 beta (IL-1β), and recruiting immune cells from the peripheral circulation [10, 11].

Current therapeutic strategies primarily utilize pyrimethamine, sulfadiazine, and spiramycin to inhibit *T. gondii* proliferation, or traditional Chinese medicine components such as ginsenosides to suppress inflammatory responses [12]. These agents exert antiinflammatory effects by targeting the NLRP3 inflammasome signaling pathway within microglia [12]. However, this approach demonstrates limited efficacy against chronic cyst stages and presents several drawbacks, including significant side effects and incomplete parasite clearance [13]. Although previous studies have made substantial progress in elucidating the neuropathogenic mechanisms of *T. gondii* infection, single-target research paradigms have constrained their findings in revealing the impact of infection on specific host tissues. Therefore, elucidating the profile of CNS-associated small molecule metabolic alterations, and the brain metabolic reprogramming upon *T. gondii* infection, is essential.

Following invasion, *T. gondii* continuously ingests nutrients from their host cells, while the host simultaneously initiates defense and adaptation mechanisms to maintain cellular homeostasis [14]. This bidirectional interaction inevitably leads to modifications in host metabolic products. Previous studies have demonstrated that *T. gondii* modulates host metabolites to promote parasite replication, consequently altering host cell metabolism [15], which modifies neurotransmitter levels in the mouse cerebral cortex [16]. Metabolomics has emerged as a valuable approach for investigating potential therapeutic targets against *T. gondii* infection [17]. Although the brain maintains its immune-privileged status through the selective permeability of the blood–brain barrier (BBB) and the immune surveillance network mediated by glial cells, its metabolic homeostasis imbalance reflects systemic pathophysiological changes [18]. However, we lack a comprehensive understanding of the dynamic reprogramming patterns and regulatory networks of the small molecule metabolites in CNS during *T. gondii* infection.

Metabolic reprogramming is a cellular process to dynamically and precisely regulate its metabolic network in response to the disruptions in internal microenvironmental homeostasis, leading to the reconstruction of metabolic product profiles [19]. In host–pathogen interactions, metabolic reprogramming exhibits dual regulatory characteristics. For example, macrophages can switch metabolic phenotype from mitochondrial β-oxidation to aerobic glycolysis (classic Warburg effect), and undergo cholesterol metabolic reprogramming, enhancing their antimicrobial capabilities [20]. However, metabolic hijacking is observed in human immunodeficiency virus (HIV) infection with antiretroviral therapy alone, while controlling viral load, it fails to reverse T-cell mitochondrial metabolic abnormalities and glutamine addiction [21]. Multi-omics studies demonstrate that *T. gondii* infection induces metabolic reprogramming in murine dendritic cells, characterized by increased arginine degradation and aerobic glycolysis [22]. *Toxoplasma gondii* infection also involves reprogramming of amino acid and lipid metabolism, with various lipid-related metabolites such as arachidonic acid (AA), docosahexaenoic acid, and oleic acid identified as potential infection-responsive metabolites [15]. Cholesterol metabolic reprogramming constitutes a highly dynamic and complex regulatory system encompassing cholesterol biosynthesis, efflux, esterification, transport, and oxidative metabolism [23]. Glial cell polarization can induce metabolic reprogramming, including phenotypic switching and comprehensive regulation of intracellular metabolic networks, to adapt to energy fluctuations caused by hypoxia, energy deficiency, and oxidative stress [24].

25-Hydroxycholesterol (25-HC) is an oxysterol generated through the enzymatic action of cholesterol-25-hydroxylase (Ch25H) on cholesterol, representing a key metabolite in cholesterol metabolism [25]. It can cross the blood–brain barrier (BBB) to regulate lipid metabolism within the CNS [26]. 25-HC primarily exerts antiviral and antibacterial effects by inhibiting viral replication, enhancing IL-6-mediated immune responses, and facilitating immune cell recruitment, thereby contributing to host defense and immune homeostasis maintenance [27]. 25-HC can also function as an inflammatory mediator, promoting microglial IL-1β secretion through an ApoE-4-dependent mechanism, leading to neuroinflammation [28]. Its biological functions vary significantly across different cell types, tissues, and organs. Emerging evidence suggests that 25-HC can suppress sterol regulatory element-binding protein (SREBP) synthesis, thereby reducing IL-1β production [29]. In brain tissue during pathogen infection, immune cells combat infection through inflammatory cytokines secretion. However, 25-HC may regulate these immune responses as an inflammatory mediator. These multifaceted roles make 25-HC a potential therapeutic target in infectious diseases and metabolic disorders.

In parasite’s infection, host cell cholesterol metabolism regulation is often overlooked in parasite–host interaction studies [30]. Therefore, elucidating metabolomic changes in brain tissue following *T. gondii* infection, and exploring the role of the key metabolite 25-HC in anti-*T. gondii* infection, could provide insights into treating *T. gondii* -induced brain damage. We use metabolomics to comprehensively profile host brain tissue post-*T. gondii* infection, identifying infection-induced metabolic pathway disruptions and key points in the parasite’s manipulation of host metabolic networks for immune evasion. Using microglial cells (the BV2 cell line) as a model, the impact of *T. gondii* infection on cholesterol metabolism was investigated. Subsequently, the central role of cholesterol metabolism in the pathogenesis of toxoplasmosis was uncovered, providing innovative strategies for the diagnosis, treatment, and prevention of toxoplasmosis.

## Methods

### Animal ethics

All animal experiments involved in this study have been approved by the Ethical Review Committee of Southern Medical University, and the license number for the use of experimental animals is SCXK(Su)2023-0009; the experimental ethics application number is SMUL202308003.

### Construction of the infection model

Specific pathogen-free (SPF) male sv129-WT mice (six per group), aged 8 weeks, were orally infected with 12 cysts of the ME49 strain of *T. gondii *[31]. Control mice were administered an equal volume of phosphate buffer solution (PBS) via gavage. Mice were euthanized at 9 days post-infection (DPI), and brain tissues were collected, rinsed once with 0.9% saline, and rapidly stored in liquid nitrogen for metabolomic detection.

### Cell culture and parasite maintenance

Human foreskin fibroblast (HFF) cells and BV2 cells (Microglial Cell Line) were cultured in Dulbecco's Modified Eagle Medium (DMEM) supplemented with 10% fetal bovine serum (FBS), 10,000 U/mL penicillin, and 10,000 μg/mL streptomycin, and maintained at 37 °C in a humidified atmosphere containing 5% CO_2_. The tachyzoites of *T. gondii* ME49 strain were propagated in HFF cells and cultured in DMEM containing 1% FBS, penicillin, and streptomycin under identical incubation conditions.

### LC–MS-based metabolomics

Metabolites were extracted from brain tissues using tissue extracts, chromatography was performed using an ultra-high performance liquid chromatography (LC) system, Thermo Vanquis [32]; metabolites were detected using a mass spectrometry (MS) detector, Thermo Orbitrap Exploris 120; and data were collected using an electrospray ion source (ESI). The spray voltages were 3500 V and −2500 V for positive and negative ion modes, respectively [33].

LC–MS raw data were converted to mzXML file format using the MSConvert tool in the Proteowizard package. Peak detection, peak filtering, and peak alignment were performed using the XCMS package in R to generate the quantitative list of substances. Regression correction was performed using quality control (QC) samples to reduce systematic errors, and substances with coefficients of variation greater than 30% in QC samples were removed for quality control [33].

We used the Benjamini–Hochberg method for correcting *P*-values for multiple hypothesis testing (FDR). The difference in expression of the differential metabolite was considered statistically significant only when the corrected *P*-value was less than 0.05 and the VIP value was greater than 1.0.

Differential metabolites were identified through comprehensive analysis using established metabolic databases, including the Human Metabolome Database (HMDB;http://www.hmdb.ca/spectra/ms/search), LIPID MAPS® Structure Database (LipidMap;http://www.lipidmaps.org), and the Kyoto Encyclopedia of Genes and Genomes (KEGG;http://www.genome.jp/kegg/). Metabolite identification was performed by systematic searching, comparison, and annotation of mass spectrometry data against these publicly available metabolite profile databases [34].

### RNA extraction and quantitative reverse transcription PCR

Total RNA was extracted using TRIzol reagent (Invitrogen, Thermo Fisher Scientific) and quantitative reverse transcription PCR (qRT-PCR) was conducted using HiScript III All-in-one RT SuperMix Perfect for qPCR (Vazyme, Nanjing, China). Real-time PCR detection of target genes was performed using Hieff^®^ qPCR SYBR Green premix (Low Rox Plus) (Yeasen, Shanghai, China) on a QuantStudio 6 real-time fluorescent quantitative PCR system (Thermo Fisher Scientific). Following reaction completion, raw data were analyzed using the 2^(−ΔΔCt) method to determine relative gene expression levels, with GAPDH serving as the internal reference gene for normalization. The experiments were divided into infected and control groups, brain tissue samples were collected and tested at 9 DPI, and cell samples were collected and tested 24 h after infection. All experiments were independently repeated in triplicate.

### ELISA for detection of 25-HC

After 24 h of infection with *T. gondii* ME49, the medium and cell lysate of BV2 cells were collected and centrifuged at 3000 g for 10 min. The concentration of 25-HC was quantified using an enzyme-linked immunosorbent assay (ELISA) kit (Jingmei Biotech, China).

### CCK8 assay to determine the effect of 25-HC on BV2 cell proliferation activity

The Cell Counting Kit-8 (CCK8) assay (TransGen Biotech, China) was utilized to evaluate the effect of 25-HC on the proliferation activity of BV2 cells, aiming to determine the optimal concentration for subsequent experiments. BV2 cells were seeded into a 96-well plate at a density of 50,000 cells/mL. Following a 24 h incubation period, the initial medium was discarded, and 25-HC was diluted in DMEM medium containing 1% FBS to final concentrations of 0, 1, 5, 10, 50, and 100 μg/mL. Control wells included 100 μL of DMEM as a blank control and 100 μL of DMEM with BV2 cells as a negative control. After 24 h of incubation, 10 μL of CCK8 solution was added to each well, followed by further incubation at 37 °C for 1 h. The absorbance was then measured at 450 nm using a microplate reader. Each concentration was tested in triplicate.

### Immunofluorescence assay

For the invasion assay, cells were fixed with 4% paraformaldehyde at room temperature (RT) for 15 min, followed by blocking with 10% bovine serum albumin (BSA) blocking buffer for 1 h. The cells were then incubated overnight at 4 °C with a primary antibody solution of rabbit anti-*Toxoplasma gondii* (ab138698, Abcam, China) diluted in 10% BSA blocking buffer. After washing with PBS, the cells were incubated with a secondary antibody solution of Alexa Fluor 488 goat anti-rabbit (Thermo Fisher Scientific, USA) at 37 °C for 1 h. Cells were then permeabilized with 0.5% Triton X-100 in the dark at RT for 10 min and blocked again with 10% BSA blocking buffer for 1 h. The cells were then incubated overnight at 4 °C with the rabbit anti-*Toxoplasma gondii* primary antibody solution, followed by incubation with a secondary antibody solution of Alexa Fluor 594 goat anti-rabbit (Thermo Fisher Scientific, USA) at 37 °C in the dark for 1 h. After washing three times with PBS buffer, the coverslips were carefully removed in a dark biosafety cabinet and mounted using 4',6-diamidino-2-phenylindole (DAPI) mounting medium.

For the proliferation assay, cells were fixed with 4% paraformaldehyde for 15 min, permeabilized with 0.5% Triton X-100 at RT for 10 min, and blocked with 10% BSA blocking buffer for 1 h. The cells were then incubated overnight at 4 °C with a primary antibody solution of rabbit anti-*Toxoplasma gondii*, followed by incubation with a secondary antibody solution of Alexa Fluor 488 goat anti-rabbit at 37 °C in the dark for 1 h. After washing three times with PBS buffer, the coverslips were carefully removed in a dark biosafety cabinet and mounted using DAPI mounting medium.

### Comparison of *T. gondii* invasion and proliferation capabilities in BV2 cells

BV2 cells were seeded in 12-well plates. To assess the direct effect of 25-HC on ME49, the ME49 strain was preincubated with D1 medium containing 10 µg/mL 25-HC for 2 h before invading BV2 cells to evaluate invasion efficiency.

To evaluate the indirect effect of 25-HC on ME49, BV2 cells were preincubated with D1 medium containing 10 µg/mL 25-HC for 2 h. For the invasion assay, a multiplicity of infection (MOI) of 3 was used, while for the proliferation assay, a MOI of 1 was applied. After 1 h, the cells were washed with PBS. The ratio of the number of invading *T. gondii* in the nucleus to the total number of *T. gondii* was calculated for the invasion assay, and the number of *T. gondii* in each parasite vesicle (PV) was calculated for the proliferation experiment for 10 random fields of view. The number of parasites per PV and the average number of parasites per PV were subsequently counted.

### Transcriptome sequencing and bioinformatics analysis

Total RNA was extracted using TRIzol reagent, and mRNA was enriched using magnetic beads containing Oligo (dT) to synthesize cDNA and prepare the sequencing library. Single-stranded circular DNA was amplified into DNA nanoballs through rolling circle replication, followed by sequencing on the DNBSEQ platform. Paired-end 150 (PE150) sequencing generated two reads, each 150 bp in length. Pathway enrichment analysis was performed using KEGG [35]. The experiment was divided into 10 µg/mL 25-HC pretreated and control groups and samples were collected for testing 24 h after infection.

### Statistical analysis

Data were analyzed and visualized using SPSS 20 and GraphPad Prism 9.5. All experiments were conducted in triplicate, and the results are presented as the mean ± standard error of the mean (SEM). Statistical significance was assessed using Student’s *t*-test or one-way analysis of variance (ANOVA). A *P*-value of less than 0.05 was considered statistically significant.

## Results

### Metabolomic analysis of brains from WT and ME49-infected mice

**Fig 1.**
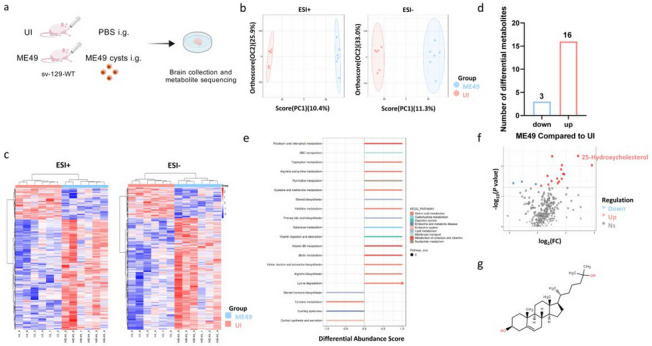
Impact of *T. gondii* infection on small molecule metabolites in brain tissue detected by untargeted metabolomic sequencing. **a** Schematic diagram of the experimental design. **b** OPLS-DA analysis of brain tissues from the two groups of mice at 9 DPI. **c** Clustering heatmap of differential metabolites in positive and negative ion modes. **d** Number of differentially expressed metabolites detected, with a fold change ≥ 10 (i.e., |Log2FC|≥ 2) and a *P*-value ≤ 0.05. **e** KEGG pathway analysis of the differentially expressed metabolites. **f** Volcano plot showing differential metabolites, highlighting representative cholesterol metabolites. **g** Structure and molecular formula of 25-hydroxycholesterol (25-HC)

To elucidate the impact of *T. gondii* infection on brain tissue metabolites, brain tissue samples collected at 9 days post-infection (DPI) were subjected to comprehensive targeted metabolomic sequencing (Fig. 1a). Orthogonal partial least squares-discriminant analysis (OPLS-DA) revealed that in positive ion mode, *R*^2^*X* = 0.628, *R*^2^*Y* = 0.999, and* Q*^2^ = 0.277; in negative ion mode, *R*^2^*X *= 0.568, *R*^2^*Y *= 0.991, and* Q*^2^ = 0.287. The *R*^2^*Y*-values approaching 1 indicate that the model effectively distinguishes between the two groups, demonstrating that *T. gondii* infection induces significant alterations in the metabolic profile of mouse brain tissue (Fig. 1b). Clustering heatmaps of differential metabolites in both positive and negative ion modes further confirmed distinct metabolic phenotypes between the infected and control groups (Fig. 1c).

Compared with uninfected mice, 19 differentially expressed metabolites were identified in the brain tissue of *T. gondii*-infected mice (Table 1). Specifically, 16 metabolites were significantly upregulated, while 3 metabolites were significantly downregulated (Fig. 1d). The differentially regulated metabolites were primarily enriched in two major signaling pathways: amino acid metabolism and cholesterol metabolism. These included tryptophan metabolism and arginine biosynthesis, which are closely related to *T. gondii* growth, as well as cholesterol metabolism pathways such as primary bile acid biosynthesis, steroid biosynthesis, and cortisol synthesis and secretion. These pathways were significantly upregulated post-infection, collectively indicating cholesterol metabolism dysregulation (Fig. 1e).

**Table 1:** List of differential metabolites in brain tissue of mice infected with *T. gondii*

Metabolites	FC	*P*-value	VIP	mz	rt	KEGG	KEGG pathway
Ergocalciferol	14.19	0.01	2.23	397.20	462.60	C05441	Steroid biosynthesis
25-Hydroxycholesterol	7.25	0.00	2.36	402.21	232.30	C15519	Primary bile acid biosynthesis
(R)2,3-Dihydroxy-3-methylvalerate	3.66	0.02	2.09	148.08	48.00	C06007	Valine, leucine, and isoleucine biosynthesis
1-Methylhistidine	3.52	0.00	2.49	170.09	43.00	C01152	Histidine metabolism
N-Acetylglutamic acid	3.50	0.02	2.02	188.06	41.50	C00624	Arginine biosynthesis
Pyridoxal	3.30	0.01	2.25	166.05	68.50	C00250	Vitamin B6 metabolism
5'-Methylthioadenosine	3.26	0.03	2.00	298.10	164.00	C00170	Cysteine and methionine metabolism
Galactitol	3.17	0.01	2.16	182.09	42.30	C01697	Galactose metabolism
Pimelic acid	2.84	0.03	1.92	159.97	88.80	C02656	Biotin metabolism
5-Aminopentanoic acid	2.68	0.04	1.81	116.93	113.70	C00431	Lysine degradation
Gingerol	2.60	0.03	1.95	293.18	466.80	C10462	Stilbenoid, diarylheptanoid, and gingerol biosynthesis
N-Acetylneuraminic acid	2.04	0.00	2.35	310.11	60.90	C19910	–
(6Z)-Octadecenoic acid	1.97	0.03	1.92	282.28	636.60	C08363	–
Porphobilinogen	1.69	0.04	1.83	226.18	644.90	C00931	Porphyrin metabolism
Deoxyuridine	1.62	0.02	2.05	228.20	645.20	C00526	Pyrimidine metabolism
Oxoadipic acid	1.19	0.04	1.74	141.07	560.30	C00322	Lysine biosynthesis
(5-L-Glutamyl)-L-glutamate	0.91	0.04	1.89	277.10	52.00	C05282	–
Cortexolone	0.46	0.03	1.92	347.23	355.60	C05488	Steroid hormone biosynthesis
Hydroquinone	0.30	0.04	1.89	110.02	298.00	C00530	Tyrosine metabolism

Only metabolites showing significant upregulation or downregulation in the comparison between the two groups are displayed. *FC* fold change, *m/z* mass-to-charge ratio, *rt* retention time

Notably, as shown in the volcano plot (Fig. 1f), one oxysterol, 25-hydroxycholesterol (25-HC, Fig. 1g) in the primary bile acid biosynthesis pathway was significantly upregulated. Previous studies have shown that 25-HC can act as an inflammatory mediator, promoting IL-1β secretion in microglia via an ApoE-4-dependent mechanism, thereby triggering neuroinflammation [28]. Therefore, we further investigated the mechanisms by which cholesterol metabolism and the associated differential metabolite 25-HC contribute to resistance against *T. gondii* infection.

### *T. gondii* infection upregulates the transcription level of CH25H in the brain

**Fig 2.**
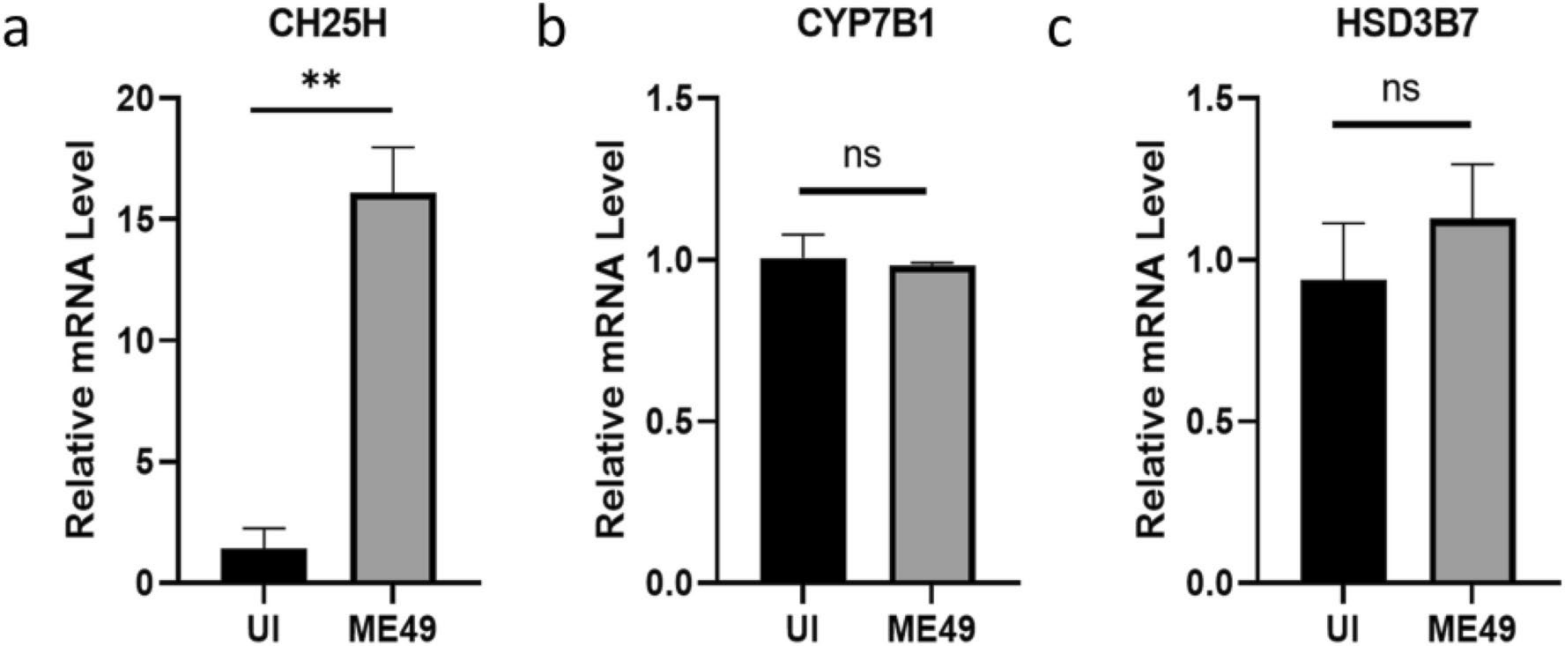
*T. gondii* infection upregulates the transcription level of CH25H in the brain. In the brains of mice infected with ME49, the transcription level of CH25H (**a**) was significantly higher than that in the brains of uninfected mice. There was no significant difference in the transcription levels of CYP7B1 (**b**) and HSD3B7 (**c**) between the two groups of mice. Statistical analysis was performed with unpaired two-tailed Students t-test. **p<0.01, ns: no significant difference.

To elucidate the molecular mechanism underlying the elevated levels of 25-HC induced by *T. gondii* infection and its association with the cholesterol metabolic network, we examined the transcription levels of key genes involved in the cholesterol oxidation pathway in the brain tissues of acutely infected mice [36]. The results indicated that, compared with the uninfected group, the transcriptional level of CH25H was significantly upregulated in the brain tissues of acutely infected mice (Fig. 2a). However, the transcription levels of downstream genes responsible for oxysterol metabolism, CYP7B1 and HSD3B7, showed no significant changes (Fig. 2b, c). This gene expression profile is highly consistent with the metabolomic analysis results, which observed a significant increase in 25-HC levels, further confirming that *T. gondii* infection activates the CH25H-mediated cholesterol oxidation pathway.

### *T. gondii* infection promotes 25-HC secretion by glial cells

**Fig 3.**
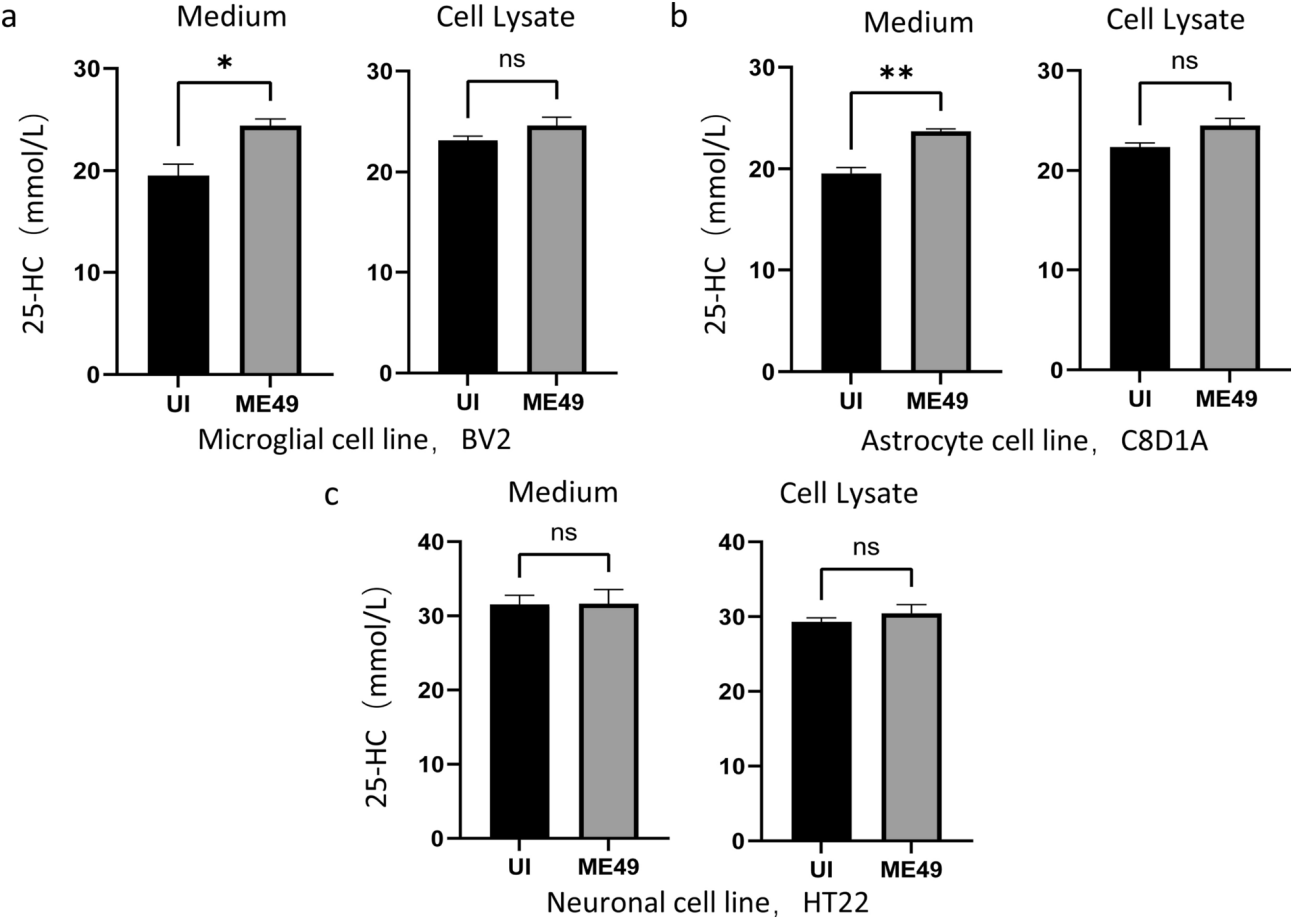
*T. gondii* infection promotes glial cells to secrete 25-HC. The levels of 25-HC in the medium and cell lysate of microglia (**a**), astrocytes (**b**), and neuron (**c**) were detected by ELISA. Statistical analysis was done by unpaired two-tailed Student’s *t*-test. *P < 0.05, **P < 0.01, *ns* no significant difference

To clarify the cellular source of the differentially expressed metabolite 25-HC and its secretion characteristics, we examined the production and secretion of 25-HC in the major cell types of brain tissue (including microglia, astrocytes, and neuronal cells) after *T. gondii* infection by ELISA. The results (Fig. 3a, b) showed that *T. gondii* infection promoted the secretion level of 25-HC in the culture supernatants of BV2 cells and astrocytes, whereas no significant change in the level of 25-HC was detected in the cell lysates of the corresponding cells, and the neuronal cells did not show significant 25-HC secretion ability before and after the infection (Fig. 3c). The above results indicated that *T. gondii* infection could specifically activate the 25-HC secretion function of neuroglial cells (including microglia and astrocytes), suggesting that glial cells play a key role in the reprogramming of cholesterol metabolism in brain tissue induced by *T. gondii* infection.

### *T. gondii* infection reprograms cholesterol metabolism in microglia

**Fig 4.**
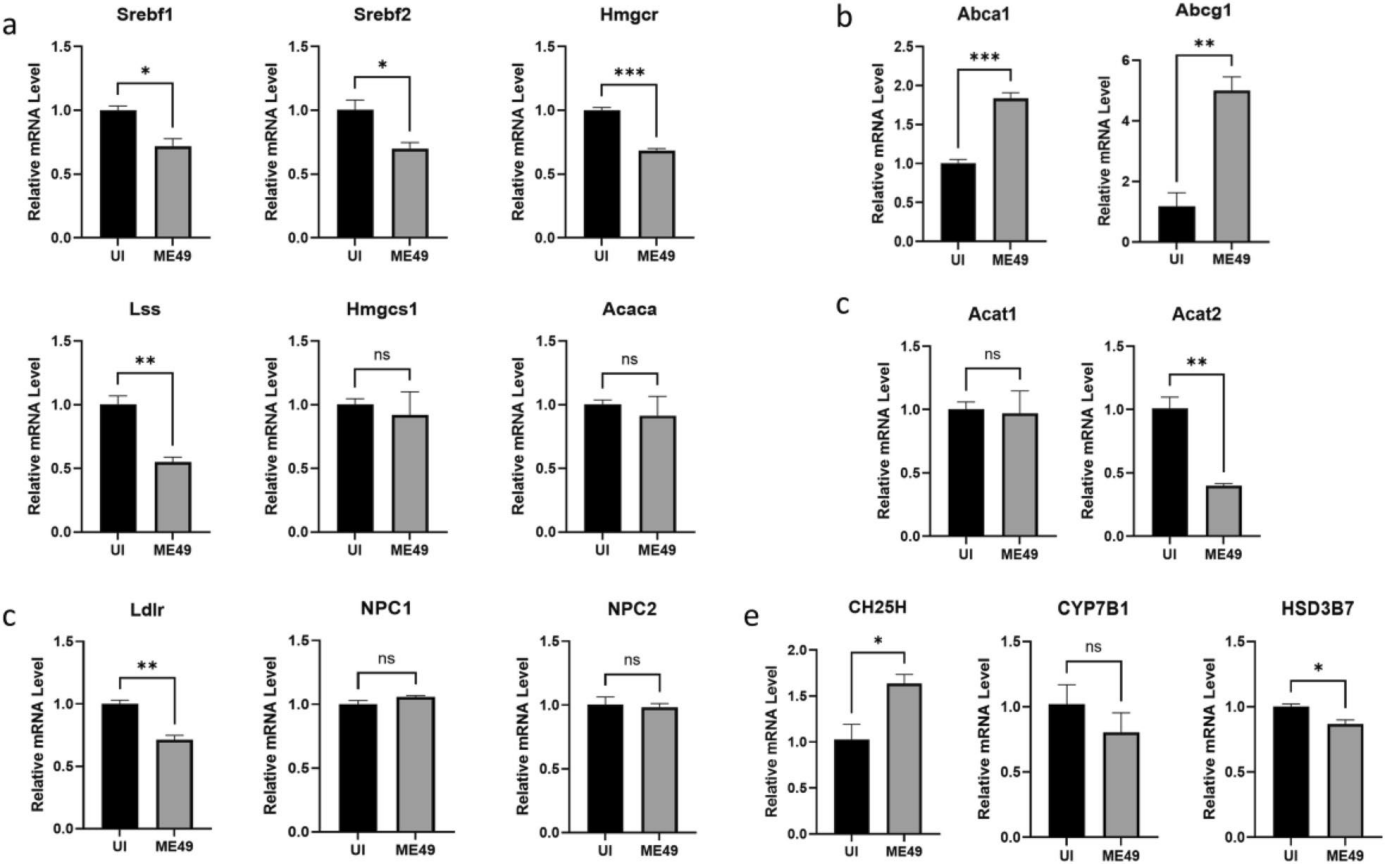
*T. gondii* infection reprograms cholesterol metabolism in microglia. **a** The transcription levels of Srebf1, Srebf2, Hmgcr, and Lss in the ME49-infected group were significantly lower than those in the uninfected group. The transcription levels of Hmgcs1 and Acaca showed no significant differences between the two groups. **b** The transcription levels of Abca1 and Abcg1 in the ME49-infected group were significantly higher than those in the uninfected group. **c** The transcription level of Acat2 in the ME49-infected group was significantly lower than that in the uninfected group. The transcription level of Acat1 showed no significant difference between the two groups. **d** The transcription level of Ldlr in the ME49-infected group was significantly lower than that in the uninfected group. The transcription levels of NPC1 and NPC2 showed no significant differences between the two groups. **e** The transcription level of CH25H in ME49-infected BV2 cells was significantly higher than that in the uninfected group. The transcription levels of CYP7B1 and HSD3B7 showed no significant differences between the two groups. Statistical analysis was done by unpaired two-tailed Student’s *t*-test. *P < 0.05, *ns* no significant difference

To comprehensively elucidate the impact of *T. gondii* infection on cholesterol metabolism in BV2 cells, we examined a series of regulatory factors involved in the reprogramming of cholesterol metabolism [37]. The results showed that compared with the uninfected group, *T. gondii* infection significantly downregulated the transcriptional levels of cholesterol synthesis enzymes in BV2 cells. This included reduced mRNA levels of sterol regulatory element-binding transcription factors Srebf1 and Srebf2, 3-hydroxy-3-methylglutaryl-CoA reductase (Hmgcr), and lanosterol synthase (Lss). In contrast, the mRNA levels of 3-hydroxy-3-methylglutaryl-CoA synthase (Hmgcs1) and acetyl-CoA carboxylase (Acaca) showed no significant changes (Fig. 4a). These findings suggest that *T. gondii* infection disrupts host cell cholesterol metabolic homeostasis by inhibiting the Srebf signaling pathway.

Simultaneously, *T. gondii* infection significantly upregulated the mRNA levels of key enzymes in the cholesterol efflux pathway, including ATP-binding cassette transporters Abca1 and Abcg1, indicating that the *T. gondii* infection activates the cholesterol efflux pathway in BV2 cells (Fig. 4b). Additionally, *T. gondii* infection significantly downregulated the mRNA level of acyltransferase 2 (Acat2), a key molecule in the cholesterol esterification pathway, but had no significant effect on its isoenzyme acyltransferase 1 (Acat1) (Fig. 4c). Regarding the cholesterol transport pathway, *T. gondii* infection significantly downregulated the mRNA level of the low-density lipoprotein receptor (Ldlr), but had no significant impact on NPC1 and NPC2, proteins responsible for intracellular cholesterol transport (Fig. 4d). This suggests that *T. gondii* infection inhibits Ldlr transcription to restrict exogenous cholesterol uptake in BV2 cells while maintaining the relative stability of the intracellular cholesterol transport system.

Finally, in the cholesterol oxidation pathway, *T. gondii* infection significantly upregulated the mRNA level of cholesterol 25-hydroxylase (CH25H), the key rate-limiting enzyme that catalyzes the conversion of cholesterol to 25-HC. However, it did not affect other key enzymes in the primary bile acid biosynthesis pathway, including oxysterol 7α-hydroxylase (cytochrome P450 family 7 subfamily B member 1, CYP7B1) and 3β-hydroxy-Δ5-C27-steroid dehydrogenase/isomerase (HSD3B7) (Fig. 4e). This selective regulatory pattern suggests that *T. gondii* infection specifically activates the CH25H-mediated cholesterol oxidation pathway, potentially limiting *T. gondii* growth within host cells by increasing 25-HC secretion. Collectively, these results reveal that *T. gondii* infection reprograms cholesterol metabolism in BV2 cells. They further suggest that *T. gondii* infection may disrupt the host cholesterol metabolic network, leading to abnormal accumulation of oxysterol metabolites such as 25-HC, thereby causing an imbalance in cholesterol metabolic homeostasis in brain tissue.

### The effect of 25-HC treatment on the invasion and proliferation ability of the ME49 strain

**Fig 5.**
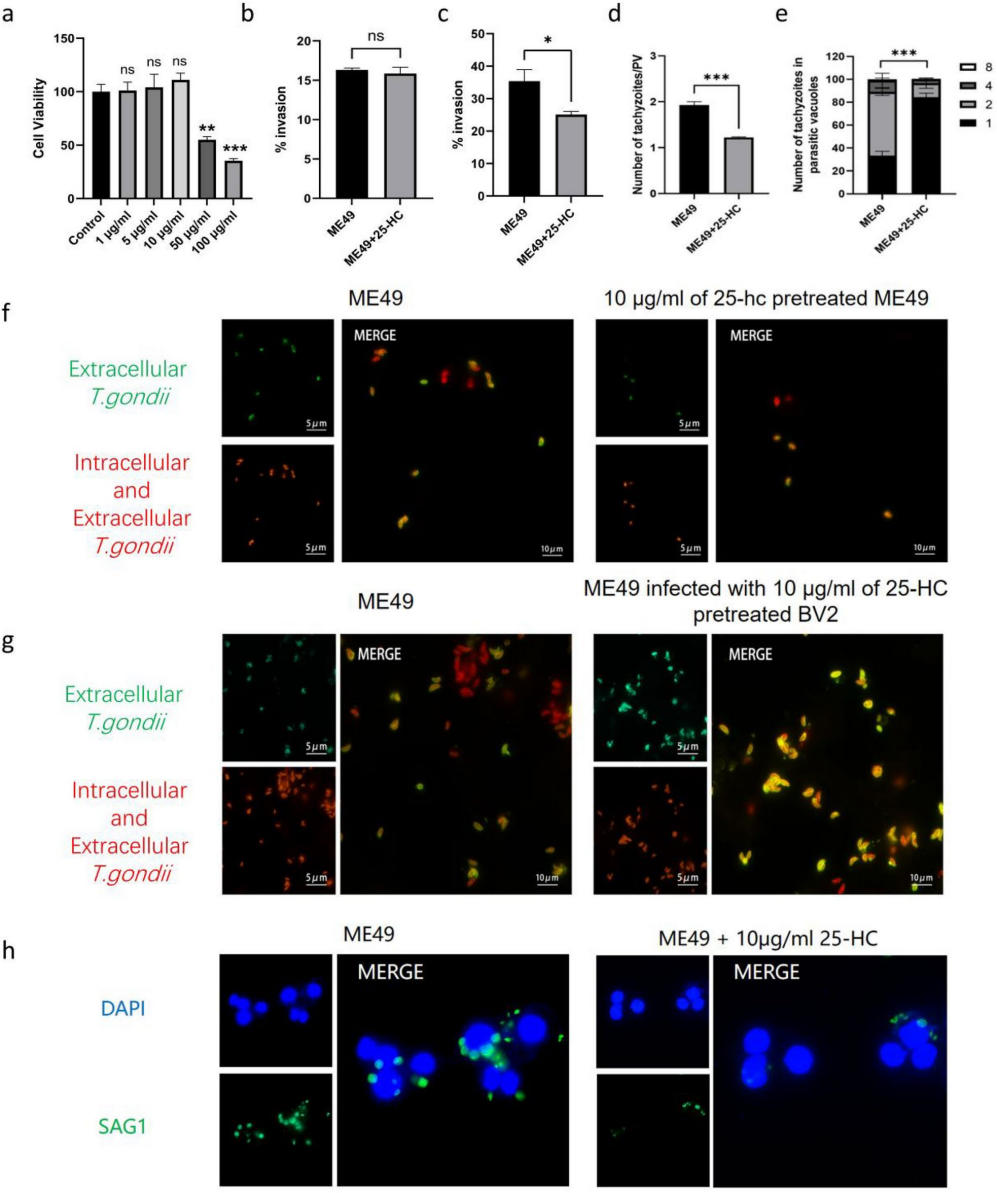
Inhibition of invasion and proliferation of ME49 strain by 25-HC treatment of BV2 cells. **a** When treated with 25-HC at concentrations ranging from 1 to 10 μg/ml, cell viability showed no significant difference compared with the control group. However, upon treatment with 25-HC at concentrations above 50 μg/ml, cell viability was significantly lower than that of the control group. **b** The treatment of ME49 with 25-HC does not affect the ability of ME49 strain to invade BV2 cells. **c** 25-HC treatment of BV2 cells reduces the invasion efficiency of the ME49 strain. **d** The number of tachyzoites within each of 100 parasitophorous vacuoles. **e** The average number of tachyzoites per parasitophorous vacuoles were calculated. **f** Detection of the invasion efficiency of ME49 strains treated with 25-HC and untreated by indirect immunofluorescence. **g** Detection of ME49 invasion into BV2 cells after 1 h of infection under conditions with and without 10 µg/ml 25-HC treatment by immunofluorescence. **h** Detection of ME49 proliferation in BV2 cells after 24 h of infection under conditions with and without 10 µg/ml 25-HC treatment by immunofluorescence. Statistical analysis was done by unpaired two-tailed Student’s *t*-test. ***P < 0.001

To further explore the biological function of 25-HC and its effects on the in vitro function of the ME49 strain, we first determined the optimal concentration of 25-HC in BV2 cells using the CCK8 assay, which was found to be 10 µg/mL (Fig. 5a). On this basis, we investigated the direct effects of 25-HC on the ME49 strain. Indirect immunofluorescence assays (IFA) were used to measure the invasion efficiency of ME49 parasites treated with 10 µg/mL 25-HC into BV2 cells. The results showed no significant difference in invasion efficiency between the treated and control groups, with rates of 15.86% and 16.33%, respectively (Fig. 5b), indicating that 25-HC has no direct effect on the invasion capability of the ME49 strain.

Subsequently, we investigated whether pretreatment of BV2 cells with 25-HC would affect the invasion of the ME49 strain. We found that BV2 cells treated with 10 µg/mL 25-HC significantly reduced the invasion efficiency of the ME49 strain, with an invasion rate of 25.11% in the treated group compared with 35.39% in the control group (Fig. 5c). Finally, to understand the impact of 25-HC treatment on the intracellular proliferation of the ME49 strain, we measured the proliferation efficiency of ME49 strain in treated and control BV2 cells. The results showed that the average number of tachyzoites per parasitophorous vacuole in the 25-HC-treated group was 1.2, significantly lower than the 1.9 in the control group (Fig. 5d). Moreover, most PV in the treated group contained only one tachyzoite (Fig. 5e), indicating a remarkable reduction in proliferation efficiency. These results demonstrated that treatment of BV2 cells with 25-HC significantly inhibits the invasion and proliferation capabilities of the ME49 strain (Fig. 5f, g, h).

### Transcriptomic effects of 25-HC treatment on ME49-infected BV2 Cells

**Fig 6.**
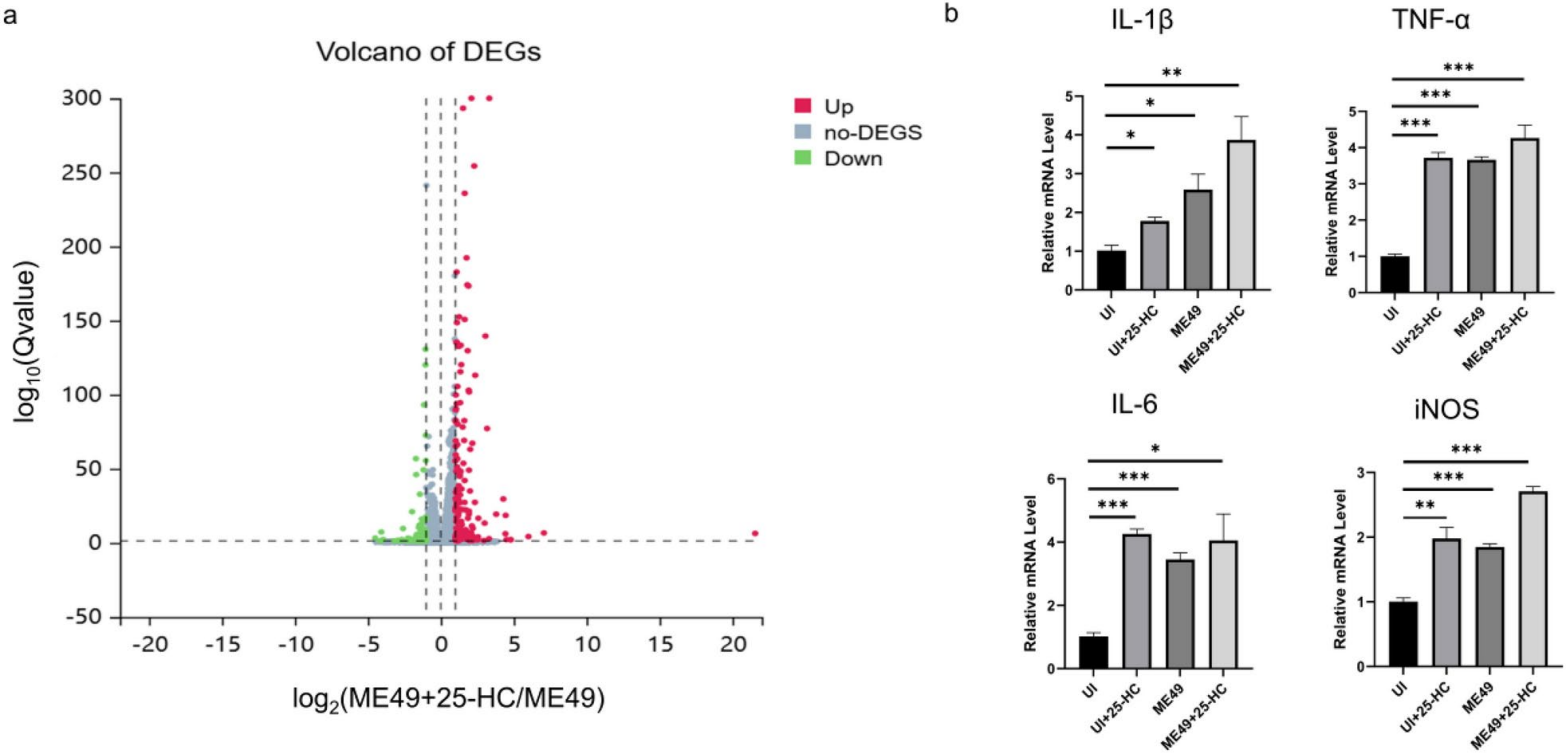
M1 polarization of BV2 cells against *T. gondii* infection. **a** Volcano map of DEGs under 25-HC treatment. **b** Both 25-HC treatment and *T. gondii* ME49 strain infection significantly upregulated the transcriptional levels of IL-1β, TNF-α, IL-6 and iNOS, with the most pronounced upregulation observed in the combined 25-HC-treated and ME49-infected group. Statistical analysis was done by unpaired two-tailed Student’s *t*-test. *P < 0.05, **P < 0.01, ***P < 0.001

To further clarify the mechanism by which 25-HC resists *T. gondii* infection, we compared the gene expression profiles of BV2 cells treated with 25-HC and infected with ME49 (ME49 + 25-HC) versus BV2 cells infected with ME49 alone (ME49) using transcriptomic analysis. It is worth noting that, as shown in the volcano plot, differentially expressed genes (DEGs) were significantly up-regulated after 25-HC treatment (Fig. 6a). The list of DEGs is presented in Table 2 (only genes related to inflammation are displayed). The results revealed that 25-HC treatment of BV2 cells led to significant differential expression of host genes across multiple signaling pathways, with DEGs primarily associated with host resistance to *T. gondii* and tissue damage repair.

**Table 2:** List of differentially expressed genes after treatment of *T. gondii*-infected BV2 cells with 25-HC

Gene ID	Gene	log_2_(ME49+25HC/ME49)	FC	*Q*-value (ME49+25HC/ME49)	KEGG pathway
330,122	Cxcl3	4.51	22.73	6.12E-03	04060: Cytokine-cytokine receptor interaction
20,310	Cxcl2	4.29	19.51	2.36E-30	04060: Cytokine-cytokine receptor interaction
20,306	Ccl7	2.57	5.95	2.41E-17	04060: Cytokine-cytokine receptor interaction
13,051	Cx3cr1	2.55	5.86	8.72E-05	04060: Cytokine-cytokine receptor interaction
56,619	Clec4e	2.29	4.90	4.75E-255	04625: C-type lectin receptor signaling pathway
56,620	Clec4n	1.86	3.64	2.29E-17	04625: C-type lectin receptor signaling pathway
20,296	Ccl2	1.64	3.11	8.22E-43	04060: Cytokine-cytokine receptor interaction
16,197	Il7r	1.64	3.11	1.73E-151	04060: Cytokine-cytokine receptor interaction
20,303	Ccl4	1.61	3.06	3.27E-83	04060: Cytokine-cytokine receptor interaction
17,474	Clec4d	1.34	2.54	3.48E-116	04625: C-type lectin receptor signaling pathway

Only differentially expressed genes related to chemokines and inflammation are shown; *FC* fold difference

On the basis of the transcriptomic analysis, the genes showing significant differences under 25-HC treatment were mainly chemokines and inflammation-related genes. Among these, C-type lectin receptors Clec4e and Clec4d can recognize pathogens and activate immune cells, triggering inflammatory responses [38]. Therefore, we examined the expression of genes associated with M1 polarization. The results showed that in BV2 cells treated with 25-HC for 2 h and infected with ME49, M1 polarization markers such as IL-1β, TNF-α, IL-6, and inducible nitric oxide synthase (iNOS) were significantly upregulated (Fig. 6b). These findings indicate that 25-HC promotes the secretion of proinflammatory cytokines in BV2 cells to resist *T. gondii* infection.

## Discussion

*Toxoplasma gondii* primarily infects hosts by the ingestion of undercooked meat containing tissue cysts or by consuming water contaminated with oocysts. Accordingly, our study specifically utilized the type II *T. gondii* ME49 strain, infecting sv129 mice via oral gavage with cysts to simulate natural infection routes. The life cycle of *T. gondii* is complex and diverse, not only involving the interconversion of tachyzoites and bradyzoites in morphology, but also requiring both asexual and sexual reproduction in intermediate or definitive hosts [39]. This characteristic drives hosts to adjust their metabolic levels to resist *T. gondii* infection during the process. In our experiments, sv129 mice infected with *T. gondii* began to show acute-phase symptoms such as weight loss, reduced appetite, and decreased activity between 7 DPI and 8 DPI, with mortality occurring between 10 DPI and 11 DPI. Therefore, in this study, we collected brain tissue samples at 9 DPI to characterize the acute infection phase. Other studies have also selected 10 DPI as a timepoint to characterize the acute infection phase and investigate transcriptional changes in brain tissue [40].

Previous studies have detected 60 differentially expressed metabolites in brain tissues at different stages of *T. gondii* infection through metabolomic analysis. These metabolites are primarily involved in pathways such as carbohydrate metabolism, lipid metabolism, and fatty acid oxidation [41]. Metabolomic analysis of the spleens of *T. gondii*-infected mice revealed 23 significantly altered metabolites during acute infection, mainly associated with lipid metabolism, amino acid metabolism, and immune responses [42]. In the liver, metabolomic analysis showed 389 significantly altered metabolites during acute infection, with the most notable changes in lipid metabolism pathways such as steroid hormone biosynthesis and primary bile acid biosynthesis [43]. The metabolic phenotypic changes induced by *T. gondii* infection exhibit tissue-specific differences. This heterogeneity may be attributed to the inherent biological characteristics of host tissue types, variations in infection doses, or differences in the selection of experimental animals. Notably, despite these inter-tissue metabolic response differences, cholesterol metabolism dysregulation induced by *T. gondii* infection is commonly observed across host tissues. This finding aligns closely with our study’s revelation that *T. gondii* infection reprogrammed host cholesterol metabolism and triggered a feedback mechanism to upregulate 25-HC secretion.

This study also found that cholesterol metabolism pathways, such as steroid biosynthesis and primary bile acid biosynthesis, were significantly upregulated. Cholesterol homeostasis is regulated by negative feedback from cholesterol itself and its derivatives, oxysterols, with oxysterols exhibiting stronger inhibitory effects on cholesterol synthesis [44]. Notably, the differentially expressed metabolite 25-HC identified in this analysis belongs to the primary bile acid biosynthesis pathway. It can promote ubiquitination and proteasomal degradation, leading to reduced levels of Hmgcr, the rate-limiting enzyme in cholesterol biosynthesis, thereby inhibiting cholesterol synthesis [45]. Previous studies have shown that *T. gondii* can interfere with fatty acid, lipid, and energy metabolism in the liver by modulating the host peroxisome proliferator-activated receptor (PPAR) signaling pathway [46]. Cholesterol metabolism dysregulation is one of the host defense mechanisms against *T. gondii* infection [47]. It has been confirmed that 25-HC can activate the NLRP3 inflammasome, causing brain inflammation [48], and has been identified as an integrin ligand capable of directly inducing proinflammatory responses in macrophages [49]. However, the mechanism by which the host regulates immune responses to induce lipid metabolism dysregulation during *T. gondii* infection remains unclear.

This study revealed that *T. gondii* infection reprogrammed the cholesterol metabolic network in BV2 cells through a dual regulatory mechanism. At the anabolic metabolism level, *T. gondii* infection downregulated the transcription levels of cholesterol synthesis enzymes in BV2 cells by inhibiting the Srebf signaling pathway. In the exogenous uptake pathway, it restricted the uptake of exogenous cholesterol by BV2 cells through suppressing Ldlr transcription, while maintaining the relative stability of the intracellular cholesterol transport system. More importantly, *T. gondii* infection specifically activates the CH25H-mediated cholesterol oxidation metabolic axis. Studies have shown that 25-HC activated CH25H expression in a Liver X receptors (LXR)-dependent manner, thereby exerting its biological functions and promoting increased secretion of 25-HC [50]. This further validated that *T. gondii* infection induced host cholesterol metabolic reprogramming and significantly upregulated 25-HC expression.

It has been reported that 25-HC accumulated in lysosomes can competitively bind to GPR155, thereby inhibiting the kinase mTORC1 and leading to the activation of AMPKα and cholesterol metabolic reprogramming [37]. Therefore, 25-HC may affect *T. gondii* invasion by regulating the lipid composition or structure of macrophage cell membranes. Additionally, *T. gondii* infection can trigger macrophages and dendritic cells to secrete IL-12 and activate natural killer cells (NK cells) to release proinflammatory cytokines such as IFN-γ. This recruits effector proteins such as immunity related GTPases (IRGs) and guanylate-binding proteins (GBPs) to the parasitophorous vacuole membrane (PVM), effectively clearing *T. gondii* from the host [51]. Thus, 25-HC may also resist *T. gondii* infection by activating the innate immune response in microglia, leading to the production of various proinflammatory cytokines. As a key intermediate product of cholesterol metabolism, 25-HC has been demonstrated to have broad antiviral effects [52]. Therefore, its inhibitory effect on *T. gondii* growth observed in this study may be related to its ability of promoting the secretion of inflammatory factors and influencing host cell membrane lipid metabolism.

Previous studies have shown that CH25H and 25-HC can reprogram macrophage metabolism and influence their polarization state, uncovering a new mechanism by which cholesterol metabolism regulates macrophage antitumor activity [53]. M1 polarization is a specific activation state of macrophages. During pathogen infection or injury, microglia can differentiate to a proinflammatory phenotype (M1 type). In this state, microglia secrete proinflammatory cytokines such as TNF-α and IL-1β, as well as reactive oxygen species, to combat pathogens or promote brain tissue repair [54]. Our study also demonstrated that 25-HC could resist *T. gondii* infection by promoting M1 polarization in host cells. On the basis of these findings, we concluded that regulating cholesterol metabolic homeostasis, as induced by *T. gondii* infection, presented a promising therapeutic approach. As a small molecule metabolite, 25-HC holds significant potential for the treatment of *T. gondii* infection.

## Conclusions

Our study is the first to characterize the metabolic changes in host brain tissue induced by *T. gondii* infection, and to identify multiple biomarkers, including 25-HC, with diagnostic and therapeutic potential. Notably, *T. gondii* infection induces host cholesterol metabolic reprogramming, activates the CH25H-mediated cholesterol oxidation pathway in the brain, and triggers a feedback mechanism that upregulates 25-HC secretion. Furthermore, 25-HC resists *T. gondii* infection by promoting M1 polarization in host cells.

## Supplementary Information

Additional file 1: The primers used in the qRT-PCR experiment

## Data Availability

Data supporting the main conclusions of this study are included in the manuscript.

## Abbreviations

CNS: Central nervous system
IFA: Immunofluorescence assay
DEGs: Differentially expressed genes
BBB: Blood–brain barrier
TNF-α: Tumor necrosis factor-alpha
IFN-γ: Interferon-gamma
25-HC: 25-Hydroxycholesterol
Ch25H: Cholesterol-25-hydroxylase
CYP7B1: Cytochrome P450 family 7 subfamily B member 1
HSD3B7: 3β-Hydroxy-Δ5-C27-steroid dehydrogenase/isomerase
Srebf: Sterol regulatory element-binding transcription factors
Hmgcr: Hydroxy-3-methylglutaryl-CoA reductase
Lss: Lanosterol synthase
Hmgcs: Hydroxy-3-methylglutaryl-CoA synthase
Acaca: Acetyl-CoA carboxylase
Acat2: Acyltransferase 2
Ldlr: Low-density lipoprotein receptor
